# Detection of interaction between biomineralising proteins and calcium carbonate microcrystals

**DOI:** 10.3762/bjnano.2.26

**Published:** 2011-04-27

**Authors:** Hanna Rademaker, Malte Launspach

**Affiliations:** 1 Institute of Biophysics, University of Bremen, Bremen, Germany

**Keywords:** biomineralisation, biomineralising proteins, Haliotis, nacre, protein–mineral interaction

## Abstract

The natural composite nacre is characterised by astonishing mechanical properties, although the main constituent is a brittle mineral shaped as tablets interdispersed by organic layers. To mimic the natural formation process which takes place at ambient conditions an understanding of the mechanism responsible for a defined microstructure of nacre is necessary. Since proteins are assumed to be involved in this mechanism, it is advantageous to identify distinct proteins interacting with minerals from the totality of proteins contained in nacre. Here, we adopted and modified a recently published approach given by Suzuki et al. [1] that gives a hint of specific protein–mineral interactions. Synthesised aragonite or calcite microcrystals were incubated with a protein mixture extracted from nacre of *Haliotis laevigata*. After incubation the mineral phase was dissolved and investigated for attached proteins. The results give a hint of one protein that seems to bind specifically to aragonite and not to calcite. The presented protocol seems to be suitable to detect mineral binding proteins quickly and therefore can point to proteins whose mineral binding capabilities should be investigated further.

## Introduction

Biological synthesised materials from various organisms such as nacreous shells have attracted the attention of many researchers from different disciplines over a number of years. Nacre as an integral part of the protective shell of some marine organisms is characterised by its astonishing mechanical properties and beautiful iridescence. Although composed only of brittle calcium carbonate mineral platelets embedded in a mechanically weak organic layer, nature has created a tough material. For recent reviews dealing with biomineralisation and especially nacre consult [2] and [3].

The fracture resistance of the whole shell and especially nacre, which consists of about 95 wt % of a brittle mineral, is achieved by a defined microstructure and a complex interaction between the mineral and the organic constituents. The microstructure of nacre can be thought of as a brick-and-mortar structure where polygonal aragonite platelets with a height of approximately 0.5 μm and a lateral extension in the micrometer range represent the bricks, whilst the organic layers around the platelets act like a kind of glue. The main toughening mechanisms in nacre include: Increasing the crack surface by crack deflection at interfaces, resistance to platelet sliding due to a rough platelet surface, platelet interlocking mechanisms and reduction of peak stresses due to crack tip blunting in the organic layer.

The organic layer between the mineral platelets comprises a chitin core with an attached protein layer. In combination with solubilised proteins, the organism is capable of producing aragonite platelets of distinct dimensions. Consequently, there must exist some kind of control mechanism for the crystal polymorph, morphology and orientation as well as crystal nucleation induction and prevention of uncontrolled crystallisation. Since proteins are assumed to play a major role in this mechanism, it would be advantageous to characterise the different proteins purified from bio-composites by their ability to bind to different minerals selectively. Such a characterisation assay or protocol is even useful for the design of peptides mimicking the biomineralisation process. It would be possible to evaluate the synthesis results of different peptides in terms of their mineral or ceramic binding capability.

Turning to proteins purified from nacre from a marine shell or of a shell forming animal, it would be helpful to determine which proteins are involved in nacre or shell formation. Recently, Suzuki and co-workers [1] described an assay to detect whether a protein binds to aragonite or calcite microcrystals specifically. Here, we wish to report how to modify the protocol given by Suzuki et al. and apply it to a mixture of soluble proteins extracted from nacre from *Haliotis laevigata*. Briefly, the experimental approach comprises the following steps: Growth of aragonite or calcite microcrystals with evaluation of the purity of the mineral phase, demineralisation of a shell from *Haliotis laevigata*, removal of the proteins from the chitin core of the remaining organic matrix from the preceding step, incubation of this protein mixture with aragonite or calcite microcrystals and finally, determination whether proteins are attached to the crystals.

## Results and Discussion

**Figure 1:**
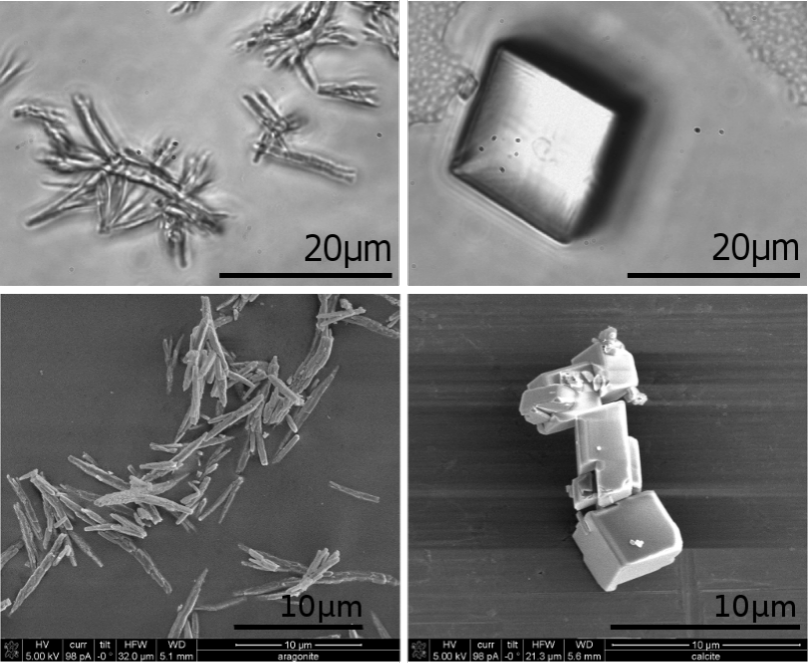
Precipitated aragonite needles (left) and calcite rhombohedrons (right). Upper: light microscopy images. Lower: scanning electron microscopy images.

**Figure 2:**
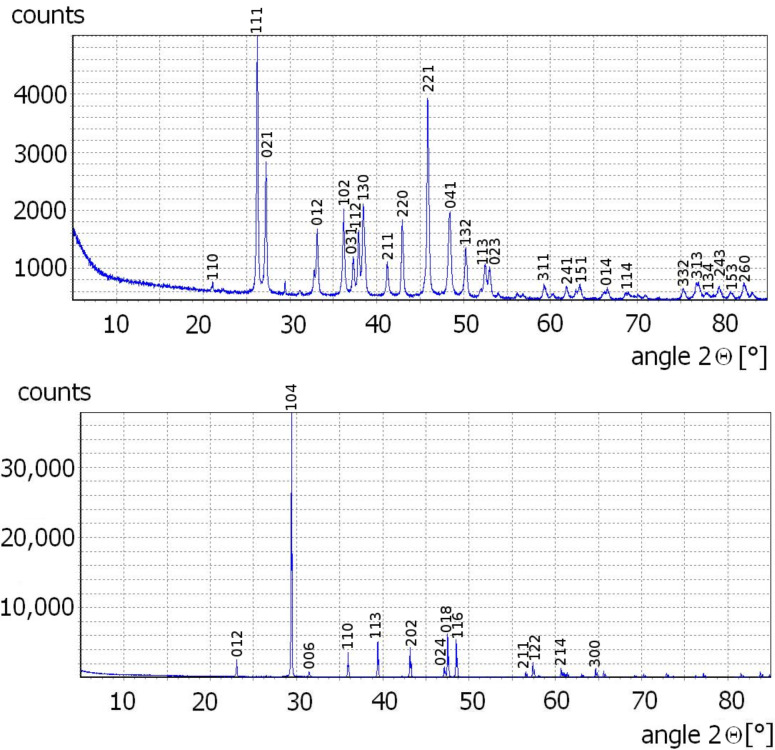
X-ray diffraction patterns of precipitated aragonite (upper) and calcite rhombohedrons (lower). Purity is 96–98 wt % for aragonite and 99–100 wt % for calcite as evaluated with the BRASS software suite [6]. Indexing according to data from [7] (aragonite) and [8] (calcite).

Light microscopy and scanning electron microscopy (SEM) images show that aragonite precipitated as needles up to 20 μm long, and calcite as rhombohedrons (Figure 1) with an edge length of about 20 μm. The purity of the microcrystal powder measured by X-ray diffraction (Figure 2) was 97 wt % for aragonite (the remaining 3 wt % are calcite impurities) and 100 wt % for calcite, with an uncertainty of 1 wt %. Thus, the protocol used for crystallization produced different polymorphs with reasonable purities. Determination of the specific surface area of aragonite and calcite by evaluation of light microscopy images and gas adsorption (BET method, see, e.g., [4]) both showed a roughly ten times larger specific surface area for aragonite. The actual values were different though, the BET method giving 4.07 m^2^/g for argonite and 0.40 m^2^/g for calcite, while the geometric estimate from the light microscopy images was 0.78 m^2^/g to 1.72 m^2^/g for aragonite and 0.10 m^2^/g to 0.19 m^2^/g for calcite. The values from the light microscopy images were estimated from 20 aragonite microcrystals assumed to be cylinders, neglecting top and bottom area, and ten calcite microcrystals assumed to be rhombohedrons with all twelve edges of equal length. The density for aragonite is 2.95 g/cm^3^ and 2.71 g/cm^3^ for calcite [5].

**Figure 3:**
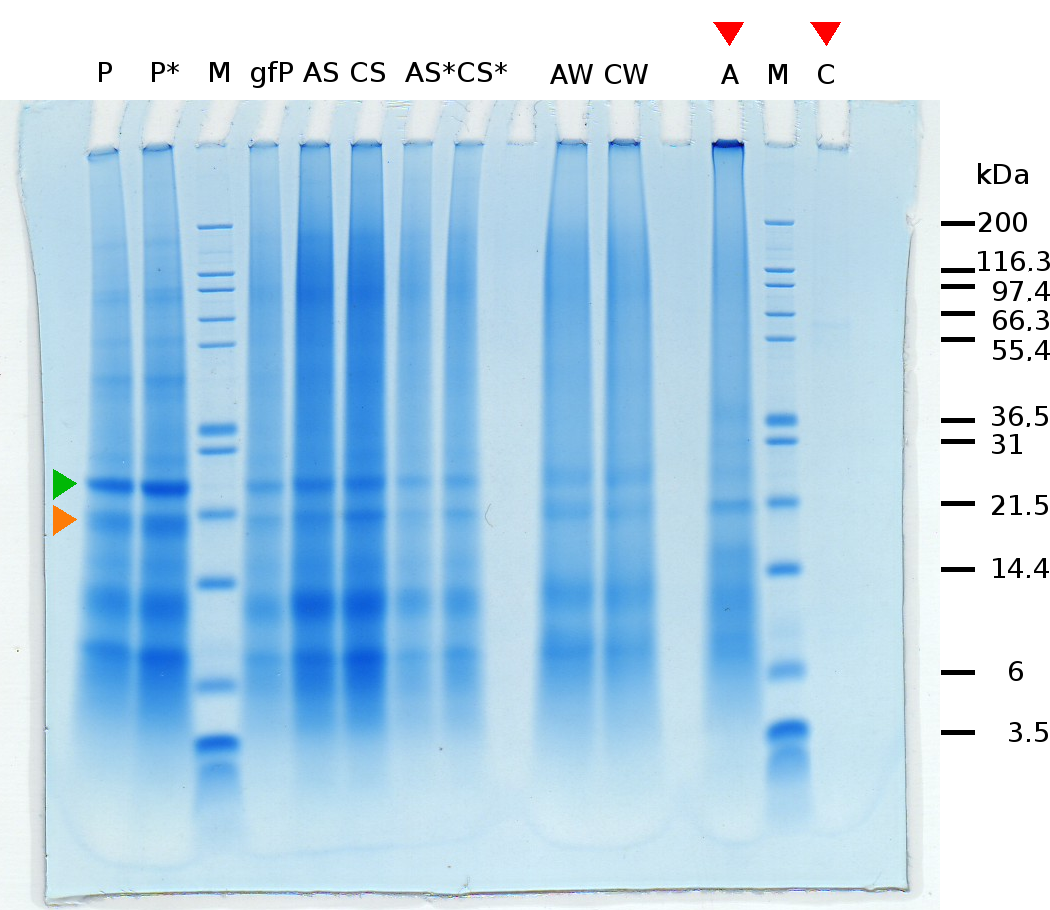
SDS-PAGE of proteins after different preparational steps as follows. **M:** marker proteins of known size. **P**, P*: protein solution of proteins that are insoluble in 6% acetic acid (chitin associated proteins), at two different concentrations. Orange marked band is less intensive than the green marked one. **gfP:** gel filtered protein solution. **AS**, **CS**, AS*, CS*: supernatant of incubated aragonite and calcite crystals, contains unbound proteins, at two different concentrations. **AW**, **CW:** combined washings of aragonite and calcite. **A**, **C:** acetic acid dissolved crystals after dialysis in sodium citrate buffer. Lane A now shows higher intensity of the orange marked band relative to green marked band, which may indicate specific binding of the orange marked protein to aragonite.

Figure 3 shows the result of the protein–crystal binding experiment. The proteins in the shell from *Haliotis laevigata* that were insoluble in 6% acetic acid were removed from the chitin core with an SDS/DTT/Tris buffer as described in the experimental section. This protein solution contained several proteins or protein fragments visible in lane P on sodium dodecyl sulfate polyacrylamide gel electrophoresis (SDS-PAGE) in Figure 3. The protein solution was gel-filtered to remove the SDS and DTT. The obtained gel-filtered protein solution (lane gfP) shows the major bands on SDS-PAGE. The gel-filtered protein solution was incubated with aragonite or calcite microcrystals. After incubation, the supernatant liquid from the aragonite or calcite microcrystals was removed and subjected to SDS-PAGE at two different concentrations (AS, AS* and CS, CS*). The crystals were washed 3 times with a NaCl/Tris solution. The washing solution was retained and combined for aragonite (AW) and calcite (CW), respectively, and also subjected to SDS-PAGE. The most dominant bands were still observable. The washed crystals were dissolved in 6% acetic acid. The proteins that were attached to the aragonite crystals (A) or to the calcite crystals (C) were obtained by removing the acetic acid by dialysis.

**Figure 4:**
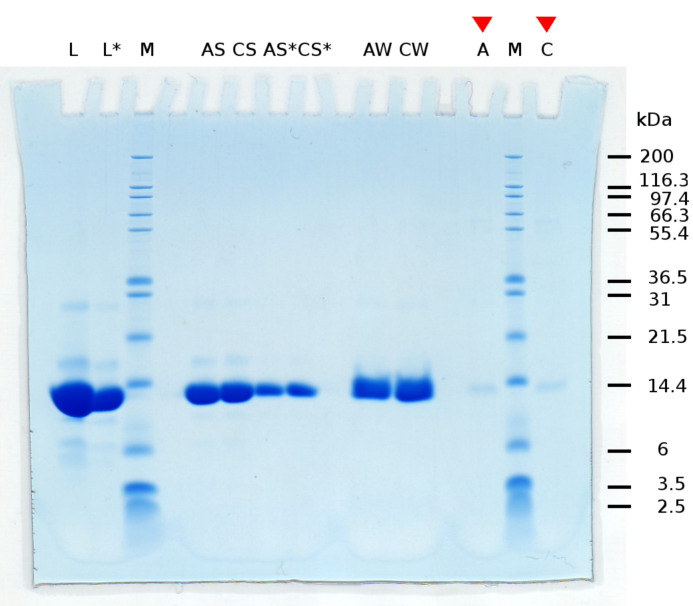
SDS-PAGE of control experiment with lysozyme. **M:** marker proteins of known size. **L**, L*: high concentrated lysozyme solution, at two different concentrations. **AS**, CS, AS*, CS*: supernatant of incubated aragonite and calcite crystals, at two different concentrations. **AW**, **CW:** combined washings of aragonite and calcite. **A**, **C:** acetic acid dissolved crystals after dialysis in sodium citrate buffer. Comparing bands in lanes A and C shows no difference in intensity.

Lanes A and C show the important difference between aragonite and calcite. In contrast to lane C, there are still proteins detectable by SDS-PAGE in lane A. This indicates that more protein was attached to the aragonite microcrystals. Generally, this could be expected due to the greater specific surface area of the aragonite microcrystals used compared to the calcite microcrystals. Nonetheless a lysozyme control experiment shows that unspecific binding cannot be the only reason for the larger amount of protein in lane A (here lane A from Figure 3). This control experiment shows the influence of the greater specific surface area of aragonite on the SDS-PAGE results, assuming unspecific binding of lysozyme to both, aragonite and calcite. A difference in intensities in lanes A and C from Figure 3 due only to unspecific binding seems unlikely, since lanes A and C (Figure 4) show no difference in intensity.

Verification of a specific binding would be most interesting, because this would be evidence that protein–mineral interaction guide polymorph selection and morphology of the calcium carbonate crystals. This would require a competitive assay. However, there is a suggestion of specific binding of one protein to aragonite in our results. Looking at the two protein bands indicated by the orange and green arrows in Figure 3 and then comparing the relative intensity of these two protein bands in lane P (entity of chitin associated proteins) and their relative intensity in lane A (aragonite associated proteins) shows an increased relative concentration of the one indicated by the orange arrow in lane A.

To estimate the detection limit for proteins in this experiment, different quantities of lysozyme were subjected to SDS-PAGE (data not shown). While for 50 ng lysozyme the band is still visible, it is not for 5 ng. So for calcite there was less than 50 ng of each protein on 10 μg crystal powder. In the protein–crystal binding experiment 15 to 20 μL of protein solution was subjected to SDS-PAGE per lane. Assuming that the detection limit for different proteins is the same, we estimate the minimum protein concentration in the subjected solution for a visible band to be 2.5 to 3.3 μg/mL.

## Conclusion

In this study a protocol given by Suzuki et al. [1] to estimate the selective mineral binding capabilities of proteins was successfully adopted and modified for nacre proteins. Firstly, the morphology, purity and specific surface area of our synthesised calcite and aragonite microcrystals was determined. After confirming the appropriate purity of the crystals, aragonite and calcite crystals were incubated with proteins that were chemically removed from the acetic acid insoluble part of nacre. The supernatant liquid of incubated aragonite and calcite crystals, washings of these aragonite and calcite samples and the acetic acid dissolved crystals were subjected to SDS-PAGE (Figure 3). The washings and the supernatant liquid contained unbound proteins. Two protein bands appeared to be present in the dissolved aragonite sample and not in the calcite sample (within the detection limit of the SDS-PAGE). It seems that the 19 kDa protein favours interactions with aragonite crystal surfaces compared to the 25 kDa one.

These two protein bands (one at approx. 19 kDa and the other at about 25 kDa) belong to the protein perlucin [10]. The corresponding fractions of purified protein showing these two bands on SDS-PAGEs had been subjected to N-terminal amino acid sequence analysis [11] in several different experiments. They always turned out to be perlucin. There might be several explanations for the different running behaviour. As perlucin has glycosylation sites there might be a subspecies of protein molecules which are glycosylated and another one which is not. There might even be the possibility that a subspecies of perlucin has saccharide oligomers bound to these sites. In MALDI-MS (matrix-assisted laser desorption/ionization mass spectroscopy) of perlucin [10] variable molecular weights of perlucin are detectable, which can be related to a 10 amino acid repeat at the C-terminus being present in various numbers of repeats. Another explanation might be that perlucin as an extracellular protein, which is incorporated in a mineral matrix, is not fully denatured by the usual procedures and therefore would have a kind of "bulky" running behaviour.

Our investigations might be a hint of specific binding of one protein to a distinct mineral polymorph. From the BET measurements and light microscopy image estimations it is known that the specific surface of the aragonite needles is approximately tenfold higher than the specific surface of calcite rhombs. Consequently, the same experimental procedure was performed with lysozyme as a control. As can be seen in Figure 4 the lysozyme bands in the dissolved aragonite and calcite sample respectively, show the same intensity indicating a non specific binding for the control protein.

We are aware that our modified protocol taken from Suzuki and co-workers can only give preliminary information about potential mineral binding proteins. Nonetheless, this fast method makes it possible to select certain proteins for further investigations. As in the case of perlucin, it is worth regarding this protein as an important model system for (theoretical) simulations of this interaction on the molecular to atomic scale and conducting further experiments.

## Experimental

As we stated several times above, the detection of interaction between biomineralising proteins and calcium carbonate microcrystals in this study is based on a modified approach from [1]. The main differences between the previously published protocol and our approach are summarised in the following. In this study, the microcrystals are freshly synthesised and not purchased. BET gas adsorption measurements were used to determine the specific surface area of the different calcium carbonate polymorphs. This information is important to distinguish between unspecific and specific binding of proteins to the different crystal polymorph surfaces. Protein solutions were concentrated by vacuum centrifugation instead of ultracentrifugation. A further difference was the composition of the washing solution for removal of proteins unspecifically attached to crystal surfaces and other proteins. Here, a slightly basic buffer solution was used to prevent crystal dissolution during washing.

### Preparation of microcrystals

Calcium carbonate microcrystals were obtained by a similar method as described by Wray and Daniels [9], using CaCl_2_ instead of Ca(NO_3_)_2_. Adding 20 mL 1.0 M CaCl_2_ to 200 mL 0.1 M Na_2_CO_3_ after pre-heating of both solutions and stirring at constant temperature results in the precipitation of aragonite, calcite and vaterite. The aragonite/calcite ratio of the precipitated polymorphs depends on reaction temperature and digestion time. For aragonite the precipitation reaction was conducted at 70 °C for 6 min, for calcite at 30 °C for 18 h. After filtration (Filter Discs (Quant.) Grade: 389, Sartorius Stedim) and washing with deionised water the microcrystals were dried at their respective reaction temperature for at least 12 h and loosened with a glass rod from time to time. The purity of the microcrystal powder was first checked with a light microscope (ZEISS Axiovert 135), then analyzed by X-ray diffraction (X’Pert Pro, PANalytical). The crystal samples were ground with a mortar and a pestle prior to the X-ray measurement. The specific surface area of the microcrystals was determined by the BET gas adsorption method (see, e.g., [4]) with N_2_ as adsorptive at a temperature of 77 K (BELsorp-miniII).

### Preparation of proteins

Shells of *Haliotis laevigata* were sand blasted (Sigg Strahlmittel, WA 70, with Al_2_O_3_) to remove the calcite layer. After 2 min in 6% NaClO and thorough washing with deionised water, the shells were dried overnight at 4 °C and crushed into pieces smaller than 0.5 cm in diameter. 40 g of one crushed shell was placed in a dialysis tube (Spectra/Por Dialysis Membrane) with molecular weight cut-off (MWCO) of 3.5 kDa and treated with 5 liters of 6% acetic acid, which was replaced two times over one week to dissolve the mineral components. The remaining flakes of acetic acid insoluble matrix were washed with deionised water and then treated for 10 min at 100 °C with 50 mL solution of 6% sodium dodecyl sulfate (SDS), 10 mM dithiothreitol (DTT), and 10 mM Tris-HCl (pH 8.0). 2.5 mL of the supernatant liquid (protein solution, P) was filtered (NALGENE syringe filters with nylon membranes, pore size: 0.2 μm) and divided into 12 parts (first one 1.0 mL, the others 0.5 mL) with a PD-10 gel column (Sephadex TMG-25 M, GE Healthcare) to remove the SDS and DTT from the solutions. Beforehand the gel columns had been equilibrated with 25 mL 10 mM Tris-HCl (pH 8.0). After determining the fractions of highest protein concentration by a Bradford Assay (Bio-Rad), fractions 5 to 9 of two gel filtration runs were combined and concentrated in a vacuum centrifuge (Savant SpeedVac SPD121P) to 250 μL (gel-filtered protein solution, gfP).

### Incubation of the microcrystals with protein solution

10 mg of the microcrystal powder were incubated with 100 μL gel-filtered protein solution for 12 h at room temperature with constant shaking. The supernatant liquid (AS for aragonite or CS for calcite) was retained and the microcrystals were washed 3 times with 100 μL solution of 240 mM NaCl, 10 mM Tris-HCl (pH 8.0). The washing solutions were retained and combined (W). After washing, the crystals (A: aragonite, C: calcite) were dissolved in 1 mL of 6% acetic acid, which was removed afterwards by dialysis (Spectra/Por Dialysis Membrane, MWCO: 3.5 kDa) in 5 mM sodium citrate buffer at pH 4.8 with 0.02% NaN_3_. After concentrating protein solution P, gel-filtered protein solution gfP, supernatant S, washing solution W and crystal solutions A and C in a vacuum centrifuge (Savant SpeedVac SPD121P) to a volume of about 20 μL, each solution was subjected to SDS-PAGE. For SDS-PAGE 12% Bis-Tris gels (Invitrogen) were used with MES (2-(*N*-morpholino)ethanesulfonic acid) as running buffer (Invitrogen). For the lysozyme control experiment, commercially available lyophilisated lysozyme was dissolved in deionised water to a concentration of 1 g/L. The microcrystals were incubated with this solution as stated above.
